# Three-Dimensional Interaction of a Large Number of Dense DEP Particles on a Plane Perpendicular to an AC Electrical Field †

**DOI:** 10.3390/mi8010026

**Published:** 2017-01-20

**Authors:** Chuanchuan Xie, Bo Chen, Jiankang Wu

**Affiliations:** 1 School of Civil Engineering and Mechanics, Huazhong University of Science and Technology, Wuhan 430074, China; d201277385@hust.edu.cn (C.X.); chbo76@hust.edu.cn (B.C.); 2 School of Urban Construction Engineering, Wenhua College, Wuhan 430074, China

**Keywords:** dielectrophoresis, particle interaction, iterative dipole moment (IDM) method, alternating current (AC) field

## Abstract

The interaction of dielectrophoresis (DEP) particles in an electric field has been observed in many experiments, known as the “particle chains phenomenon”. However, the study in 3D models (spherical particles) is rarely reported due to its complexity and significant computational cost. In this paper, we employed the iterative dipole moment (IDM) method to study the 3D interaction of a large number of dense DEP particles randomly distributed on a plane perpendicular to a uniform alternating current (AC) electric field in a bounded or unbounded space. The numerical results indicated that the particles cannot move out of the initial plane. The similar particles (either all positive or all negative DEP particles) always repelled each other, and did not form a chain. The dissimilar particles (a mixture of positive and negative DEP particles) always attracted each other, and formed particle chains consisting of alternately arranged positive and negative DEP particles. The particle chain patterns can be randomly multitudinous depending on the initial particle distribution, the electric properties of particles/fluid, the particle sizes and the number of particles. It is also found that the particle chain patterns can be effectively manipulated via tuning the frequency of the AC field and an almost uniform distribution of particles in a bounded plane chip can be achieved when all of the particles are similar, which may have potential applications in the particle manipulation of microfluidics.

## 1. Introduction

An electric force exerted on a non-conducting particle when a locally non-uniform electric field is applied is called dielectrophoresis (DEP) [1,2,3]. The particle is polarized in a non-uniform electric field, which arises from the spatial variation or time variation of the field (direct current (DC) or alternating current (AC)). The DEP force is dependent on the electric properties of the particle and fluid, the shape and size of the particle, and the frequency of the AC field. The particle is forced to move toward higher or lower electric field regions according to the difference in the electric properties of the particle and electrolyte, which are called positive and negative DEP, respectively. DEP can be easily applied to manipulate the particles and has become one of the most popular techniques in applications in biology and chemistry, such as cell separation [4,5,6], sorting [7], trapping [8,9], and assembly [10,11,12].

The electric polarization of a particle can also disturb the local electric field of the surrounding particles. When one particle is close to another, the local electric field around them becomes more non-uniform, even under a uniformly-applied field and, thus, this induces a DEP interaction force for each particle. The interaction force gets larger as the particles get closer. This electrical phenomenon is called a “particle chain”, which has been observed in other experiments [13] and is the basis of the particle assembly technique, which has been used in many applications [14,15,16,17]. Kevin et al. developed a new class of microwires by dielectrophoresis assembly from suspensions of metallic nanoparticles [14]. Shalini et al. co-assembled live cells and surface-functionalized synthetic microparticles on electrically controlled chips to yield permanent chains and one-cell-layer-thick membranes [15]. Zhou et al. used dielectrophoresis assembly to rapidly align ensembles of CdSe semiconductor nanowires near patterned microelectrodes for polarization-sensitive photodetection and biosensing applications [16]. Julia et al. developed a new microfluidic test system for liver toxicity, devising a microfluidic chip featuring cell culture chambers with integrated electrodes for the assembly of liver sinusoids by dielectrophoresis [17]. In recent years, a significant amount of effort has been devoted to the study of the DEP interactions of particles. Giner et al. [13] studied the characteristics of the particle chaining process in a mixture of polystyrene beads and yeast cells, applying a Monte Carlo simulation. Aubry et al. [18,19] developed a Lagrange multiplier–based numerical model to study the DEP interactions of particles. The DEP force is calculated by a simplified dipole method, which is sufficiently accurate when the particles are not too close. Ai et al. [20,21] and Xie et al. [22,23] performed direct numerical simulations on the interactions of two or three particles both in two-dimensional DC and AC fields by using the Maxwell stress tensor (MST) method and the arbitrary Lagrangian–Eulerian (ALE) method, and the effect of the frequency was studied. The numerical computation of the ALE method requires continuous grids moving and remeshing in the case of a larger number of particles. The MST method is the most rigorous approach to calculate the DEP forces. However, it is not practical in cases of 3D DEP interactions of a larger number of particles due to the numerical errors and the significant cost of computation. Kang et al. [24,25,26,27] performed direct numerical simulations to study the 2D interactions of five particles in both DC and AC fields by finite difference simulations. Hossan et al. [28] studied 2D interactions of a few particles in a DC field using a hybrid immersed interface-immersed boundary method. The above studies were all for a small number of 2D cylindrical particles in an electric field parallel to the particle plane. These results have deviated somewhat in comparison with the experimental results [29,30,31,32]. The DEP interactions of a large number of particles in a 3D electric field have not been well studied due to great difficulties in numerical computation.

Recently, the iterative dipole moment (IDM) method, first developed by Liu and Wu et al. [33,34,35,36], has been an effective method to simulate the DEP interactions of a large number of particles. IDM is an algebraic algorithm without complicated numerical computation for solving the Laplace equation and the Navier-Stokes equation. The accuracy, convergence, and simplicity of the IDM method have been demonstrated by comparing them with the results of the MST method in both DC and AC fields [33,34]. The motion trajectories and final chains of the particles are in good agreement with the results of the previous numerical studies and consistent with the experiments. This method has been successfully applied in the interaction of a large number of particles in 2D DC and AC fields [35,36].

In this work, the IDM model is used to simulate the DEP interactions of 3D spherical particles in a special 3D scenario where the particles are initially positioned in a plane. A uniform AC electric field is applied perpendicular to the particle plane, which is different to the prior 2D studies. No correlational numerical studies have been found so far. The phenomena of the interactions in this scenario are novel and notable, and they are distinguished from the results of previous studies. The mechanism of particle motions and the particle chains of DEP interactions of a large number of dense particles are investigated in situations of different sizes, different initial distributions, and different frequencies. Furthermore, the effect of a circular boundary to the DEP particle interactions is also investigated.

## 2. Mathematical Model

### 2.1. Physical Description

**Figure 1:**
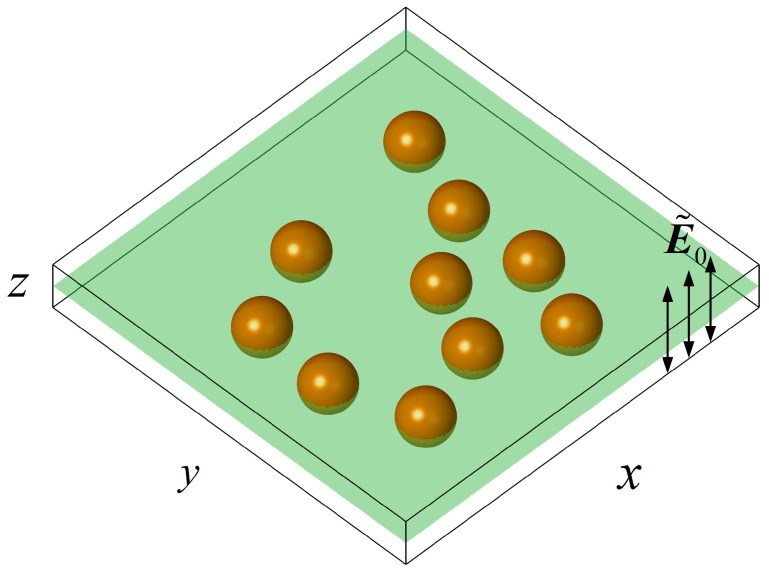
A number of particles randomly suspended on a flat chip filled with electrolyte and a uniform alternating current (AC) electric field ${\tilde{E}}_{0}$ is applied perpendicular to the chip plane.

In practical microfluidic chips the depth is much smaller than the horizontal scale. The particle motion in the chip is almost confined to a plane in cases where the particle size is of the same order as the chip depth. The 2D DEP particle interactions on a plane parallel to the electric field have been well studied. In the present study, the 3D spherical particles are randomly distributed on a plane chip, and a uniform AC electric field perpendicular to the plane is applied as shown in Figure 1.

### 2.2. The Iterative Dipole Moment Method (IDM)

The particles are located at $\left(x_{i},y_{i},z_{i})\;(i=1,2,\cdots N\right)$, respectively. When an AC electric field ${\tilde{E}}_{0}$ is applied, the particles can be polarized as dipole moments ${\tilde{p}}_{i}$, expressed as [2]

(1) $${\tilde{p}}_{i}=4\pi a_{i}^{3}\varepsilon_{f}K_{i}(\omega ){\tilde{E}}_{0}$$

where $a_{i}$ is the radius of the *i*th particle, $K_{i}(\omega )$ is the Clausius–Mossotti (C–M) factor, given by

(2) $$K_{i}(\omega )=\frac{{\tilde{\varepsilon }}_{pi}-{\tilde{\varepsilon }}_{f}}{{\tilde{\varepsilon }}_{pi}+2{\tilde{\varepsilon }}_{f}}$$

where ${\tilde{\varepsilon }}_{pi}=\varepsilon_{pi}-j\sigma_{pi}/\omega$ and ${\tilde{\varepsilon }}_{f}=\varepsilon_{f}-j\sigma_{f}/\omega$ are the complex permittivities of the *i*th particle and fluid, respectively; $j=\sqrt{-1}$ and the superscript “~” denote the complex variables; $\varepsilon_{pi}$ and $\varepsilon_{f}$ are the permittivity of the *i*th particle and fluid, respectively; $\sigma_{pi}$ and $\sigma_{f}$ are the conductivities of the *i*th particle and fluid, respectively; ω=2πf is the angular frequency of the AC electric field.

The dipole moment will induce a local electric field, called the dipole-induced field in this work, written as follows [2]

(3) $${\tilde{\varphi }}_{i}=\frac{{\tilde{p}}_{i}\cdot r_{i}}{4{\pi \varepsilon }_{f}r_{i}^{3}}=\frac{a_{i}^{3}K_{i}(\omega )\cdot ({\tilde{E}}_{0x}(x-x_{i})+{\tilde{E}}_{0y}(y-y_{i})+{\tilde{E}}_{0z}(z-z_{i}))}{r_{i}^{3}}$$

where $r_{i}(r_{i}=\left|r_{i}\right|)$ is the position vector originating from the *i*th particle location $\left(x_{i},y_{i},z_{i}\right)$ to any point (x,y,z). When the particles are close to each other, the electric field can be strikingly influenced by the local electric fields. In our work, we correct the electric field with the dipole-induced fields of the neighboring particles, written as follows:

(4) $${\tilde{E}}_{j}^{\left(1\right)}={\tilde{E}}_{0j}+\sum_{i=1,i\neq j}^{N}{\tilde{E}}_{ij}^{\left(0\right)}$$

where ${\tilde{E}}_{0j}$ is the original electric field, ${\tilde{E}}_{ij}^{\left(0\right)}$ is the dipole-induced field of the neighboring particles, and the components can be expressed as

(5) $$\begin{array}{l} {\left({\tilde{E}}_{x}\right)}_{ij}=-\frac{\partial {\tilde{\varphi }}_{i}}{\partial x}=-a_{i}^{3}K_{i}(\omega )\\ \;\times \left[\frac{{\tilde{E}}_{0x}}{r_{ij}^{3}}-\frac{3({\tilde{E}}_{0x}(x_{j}-x_{i})+{\tilde{E}}_{0y}(y_{j}-y_{i})+{\tilde{E}}_{0z}(z_{j}-z_{i}))(x_{j}-x_{i})}{r_{ij}^{5}}\right]\\ {\left({\tilde{E}}_{y}\right)}_{ij}=-\frac{\partial {\tilde{\varphi }}_{i}}{\partial y}=-a_{i}^{3}K_{i}(\omega )\\ \;\times \left[\frac{{\tilde{E}}_{0y}}{r_{ij}^{3}}-\frac{3({\tilde{E}}_{0x}(x_{j}-x_{i})+{\tilde{E}}_{0y}(y_{j}-y_{i})+{\tilde{E}}_{0z}(z_{j}-z_{i}))(y_{j}-y_{i})}{r_{ij}^{5}}\right]\\ {\left({\tilde{E}}_{z}\right)}_{ij}=-\frac{\partial {\tilde{\varphi }}_{i}}{\partial z}=-a_{i}^{3}K_{i}(\omega )\\ \;\times \left[\frac{{\tilde{E}}_{0z}}{r_{ij}^{3}}-\frac{3({\tilde{E}}_{0x}(x_{j}-x_{i})+{\tilde{E}}_{0y}(y_{j}-y_{i})+{\tilde{E}}_{0z}(z_{j}-z_{i}))(z_{j}-z_{i})}{r_{ij}^{5}}\right] \end{array}$$

where $r_{ij}=\left|r_{ij}\right|=\sqrt{{\left(x_{j}-x_{i}\right)}^{2}+{\left(y_{j}-y_{i}\right)}^{2}+{\left(z_{j}-z_{i}\right)}^{2}}$, $r_{ij}$ is pointed from *i*th particle to the *j*th particle.

The corrected field induces a new dipole ${\tilde{p}}_{i}$ and a new dipole-induced electric field ${\tilde{E}}_{ij}^{\left(1\right)}$, and then a second corrected electric field ${\tilde{E}}_{j}^{\left(2\right)}$ is obtained in the same way. The local electric field, dipole moment and the dipole-induced electric field will be iteratively corrected, until a converged electric field ${\tilde{E}}_{j}^{\left(n\right)}$ is obtained by

(6) $$\left|\frac{{\tilde{E}}_{xj}^{\left(n+1\right)}-{\tilde{E}}_{xj}^{\left(n\right)}}{{\tilde{E}}_{xj}^{\left(n+1\right)}}\right|\leq \tau ,\;\left|\frac{{\tilde{E}}_{yj}^{\left(n+1\right)}-{\tilde{E}}_{yj}^{\left(n\right)}}{{\tilde{E}}_{yj}^{\left(n+1\right)}}\right|\leq \tau ,\;\left|\frac{{\tilde{E}}_{zj}^{\left(n+1\right)}-{\tilde{E}}_{zj}^{\left(n\right)}}{{\tilde{E}}_{zj}^{\left(n+1\right)}}\right|\leq \tau$$

where τ denotes the convergence criteria of the iteration error.

When the electric field is corrected, the time-averaged DEP interaction force $F_{\mathrm{DEP}}$ can be obtained by [34]

(7) 〈FDEP〉=2πa3εfRe[K(ω)]∇|E˜|2

### 2.3. The Modified Stokes Formula of a Large Number of Dense Particles

When a particle is suspended in a still fluid, and an electric field is applied to drive the particle by dielectrophretic (DEP) force, a hydrodynamic force is passively generated on the particle due to the particle motion. Generally, the fluid flow is induced by the particle motion under a low Reynolds number (Re~10−3), and the traditional Stokes formula has been widely accepted to calculate the hydrodynamic force on a particle, written as

(8) $$F_{d}=6\pi a\eta (u-v)$$

where η is the viscosity of the fluid, u and v are the velocities of the fluid and the particle, respectively. However, when a large number of particles are densely distributed in the fluid and move close to each other, the near-field hydrodynamic hindrance must be taken into consideration. In this work, a modified Stokes formula is proposed to consider the hydrodynamic aspects.

When a spherical particle moves in a fluid, the stream function and the velocity distribution of the fluid around the particle in local Cartesian coordinates $\left(\bar{x},\bar{y},\bar{z}\right)$ located in the particle center can be expressed as [37]

(9) $$\begin{array}{l} {\bar{u}}_{x}=U(-\frac{3}{4}\frac{a}{r}+\frac{3a^{3}}{4r^{3}})\frac{\bar{x}\bar{z}}{r^{2}}\\ {\bar{u}}_{y}=U(-\frac{3}{4}\frac{a}{r}+\frac{3a^{3}}{4r^{3}})\frac{\bar{y}\bar{z}}{r^{2}}\\ {\bar{u}}_{z}=U(1-\frac{3}{2}\frac{a}{r}+\frac{a^{3}}{2r^{3}}+\frac{3}{4}\frac{a}{r}\frac{{\bar{x}}^{2}+{\bar{y}}^{2}}{r^{2}}-\frac{3a^{3}}{4r^{3}}\frac{{\bar{x}}^{2}+{\bar{y}}^{2}}{r^{2}}) \end{array}$$

where U is the magnitude of the relation velocity of the fluid and the particle, and the direction of the relative velocity is the same as $\bar{z}$ axis and $r=\sqrt{{\bar{x}}^{2}+{\bar{y}}^{2}+{\bar{z}}^{2}}$.

Suppose that a large number of particles with global Cartesian coordinates $\left(x_{i},y_{i},z_{i}\right)$ are randomly suspended in the fluid, and the relative velocity of the fluid and the particles is $u_{ri}=u_{i}-u_{pi}$, $u_{ri}=\left|u_{ri}\right|$. Two angles are introduced by $\sin\alpha =\sqrt{u_{rix}^{2}+u_{riy}^{2}}/u_{ri}$, $\cos\alpha =u_{riz}/u_{ri}$, $\sin\beta =u_{riy}/\sqrt{u_{rix}^{2}+u_{riy}^{2}}$, $\cos\beta =u_{rix}/\sqrt{u_{rix}^{2}+u_{riy}^{2}}$. The flow velocity at particle *j* disturbed by particle *i* in local Cartesian coordinates can be written as

(10) $$\begin{array}{l} {\bar{u}}_{\bar{x}ij}=u_{ri}(-\frac{3}{4}\frac{a_{i}}{r_{ij}}+\frac{3a_{i}^{3}}{4{r_{ij}}^{3}})\frac{{\bar{x}}_{ij}{\bar{z}}_{ij}}{{r_{ij}}^{2}}\\ {\bar{u}}_{\bar{y}ij}=u_{ri}(-\frac{3}{4}\frac{a_{i}}{r_{ij}}+\frac{3a_{i}^{3}}{4{r_{ij}}^{3}})\frac{{\bar{y}}_{ij}{\bar{z}}_{ij}}{{r_{ij}}^{2}}\\ {\bar{u}}_{\bar{z}ij}=u_{ri}(1-\frac{3}{2}\frac{a_{i}}{r_{ij}}+\frac{a_{i}^{3}}{2{r_{ij}}^{3}}+\frac{3}{4}\frac{a_{i}}{r_{ij}}\frac{{{\bar{x}}_{ij}}^{2}+{{\bar{y}}_{ij}}^{2}}{{r_{ij}}^{2}}-\frac{3a_{i}^{3}}{4{r_{ij}}^{3}}\frac{{{\bar{x}}_{ij}}^{2}+{{\bar{y}}_{ij}}^{2}}{{r_{ij}}^{2}}) \end{array}$$

where $\left({\bar{x}}_{ij},\;{\bar{y}}_{ij},\;{\bar{z}}_{ij}\right)$ is the coordinate of particle *j* with respect to particle *i* and $r_{ij}=\sqrt{{\left(x_{j}-x_{i}\right)}^{2}+{\left(y_{j}-y_{i}\right)}^{2}+{\left(z_{j}-z_{i}\right)}^{2}}$. The net disturbed velocity can be obtained by

(11) $$\begin{array}{l} \Delta {\bar{u}}_{\bar{x}ij}={\bar{u}}_{\bar{x}ij}-u_{rix}=u_{ri}(-\frac{3}{4}\frac{a_{i}}{r_{ij}}+\frac{3a_{i}^{3}}{4{r_{ij}}^{3}})\frac{{\bar{x}}_{ij}{\bar{z}}_{ij}}{{r_{ij}}^{2}}\\ \Delta {\bar{u}}_{\bar{y}ij}={\bar{u}}_{\bar{y}ij}-u_{riy}=u_{ri}(-\frac{3}{4}\frac{a_{i}}{r_{ij}}+\frac{3a_{i}^{3}}{4{r_{ij}}^{3}})\frac{{\bar{y}}_{ij}{\bar{z}}_{ij}}{{r_{ij}}^{2}}\\ \Delta {\bar{u}}_{\bar{z}ij}={\bar{u}}_{\bar{z}ij}-u_{riz}=u_{ri}(-\frac{3}{2}\frac{a_{i}}{r_{ij}}+\frac{a_{i}^{3}}{2{r_{ij}}^{3}}+\frac{3}{4}\frac{a_{i}}{r_{ij}}\frac{{{\bar{x}}_{ij}}^{2}+{{\bar{y}}_{ij}}^{2}}{{r_{ij}}^{2}}-\frac{3a_{i}^{3}}{4{r_{ij}}^{3}}\frac{{{\bar{x}}_{ij}}^{2}+{{\bar{y}}_{ij}}^{2}}{{r_{ij}}^{2}}) \end{array}$$

Then we can get the net disturbed velocity at particle *j* by particle *i* in global Cartesian coordinates as

(12) $$\begin{array}{l} \Delta u_{xij}=\cos\varphi (\sqrt{\Delta {\bar{u}}_{\bar{x}ij}^{2}+\Delta {\bar{u}}_{\bar{y}ij}^{2}}\cos\alpha +\Delta {\bar{u}}_{\bar{z}ij}\sin\alpha )\\ \Delta u_{yij}=\sin\varphi (\sqrt{\Delta {\bar{u}}_{\bar{x}ij}^{2}+\Delta {\bar{u}}_{\bar{y}ij}^{2}}\cos\alpha +\Delta {\bar{u}}_{\bar{z}ij}\sin\alpha )\\ \Delta u_{zij}=-\sqrt{\Delta {\bar{u}}_{\bar{x}ij}^{2}+\Delta {\bar{u}}_{\bar{y}ij}^{2}}\sin\alpha +\Delta {\bar{u}}_{\bar{z}ij}\cos\alpha \end{array}$$

By adding the net disturbed velocities of the surrounding particles to the flow field at particle *j*, the flow velocity at particle *j* can be corrected as

(13) $$\begin{array}{l} u_{xj}^{1}=u_{xj}+\sum_{i=1,i\neq j}^{N}\Delta u_{xij}^{1}\\ u_{yj}^{1}=u_{yj}+\sum_{i=1,i\neq j}^{N}\Delta u_{yij}^{1}\\ u_{zj}^{1}=u_{zj}+\sum_{i=1,i\neq j}^{N}\Delta u_{zij}^{1} \end{array}$$

where $u_{xj},u_{yj},u_{zj}$ are the initial flow field without disturbance.

The net disturbed velocities and flow field will be iteratively corrected, until a converged flow field $u_{xj}^{n},u_{yj}^{n},u_{zj}^{n}$ is obtained as

(14) $$\left|\frac{u_{xj}^{n}-u_{xj}^{n-1}}{u_{xj}^{n}}\right|\leq \tau ,\;\left|\frac{u_{yj}^{n}-u_{yj}^{n-1}}{u_{yj}^{n}}\right|\leq \tau ,\;\left|\frac{u_{zj}^{n}-u_{zj}^{n-1}}{u_{zj}^{n}}\right|\leq \tau$$

where $\tau =10^{-3}$ denotes the convergence criteria of the iteration error; then we can get the corrected fluid drag force by substituting the initial flow field with the corrected one in the Stokes formula as

(15) $$\begin{array}{l} F_{dxi}=6\pi \mu a_{i}(u_{xi}^{n}-u_{xpi})\\ F_{dyi}=6\pi \mu a_{i}(u_{yi}^{n}-u_{ypi})\\ F_{dzi}=6\pi \mu a_{i}(u_{zi}^{n}-u_{zpi}) \end{array}$$

### 2.4. The Governing Equation of the Particles and the Dimensionless Method

The motion of the particles is governed by the kinematic equation as follows

(16) $$m_{p}\frac{d^{2}r_{p}}{dt^{2}}=F_{\mathrm{DEP}}+F_{d}$$

where $m_{p}$, $r_{p}$ are the mass and the position vector of the particles, respectively.

All of the governing equations can be normalized by the characteristic length $a_{0}=5\;\mu m$, potential $\varphi_{0}=10\;V$, velocity $U_{0}=\varepsilon_{f}\varphi_{0}^{2}/\left(\eta a_{0}\right)$, time $t_{0}=a_{0}/U_{0}$, force $F_{0}=a_{0}\eta U_{0}$, and mass $m_{0}=a_{0}^{2}\eta /U_{0}$. The normalized equations are written as:

(17) FDEP∗=2πa∗Re[K(ω)]∇∗|E˜∗|2

(18) $$F_{d}^{*}=6\pi a^{*}(u^{*}-v^{*})$$

(19) $$m_{p}^{*}\frac{d^{2}r_{p}^{*}}{dt^{*2}}=F_{\mathrm{DEP}}^{*}+F_{d}^{*}$$

where ∗ denotes the dimensionless variables.

### 2.5. The Validation of the Accuracy of the Modified Stokes Formula

**Figure 2:**
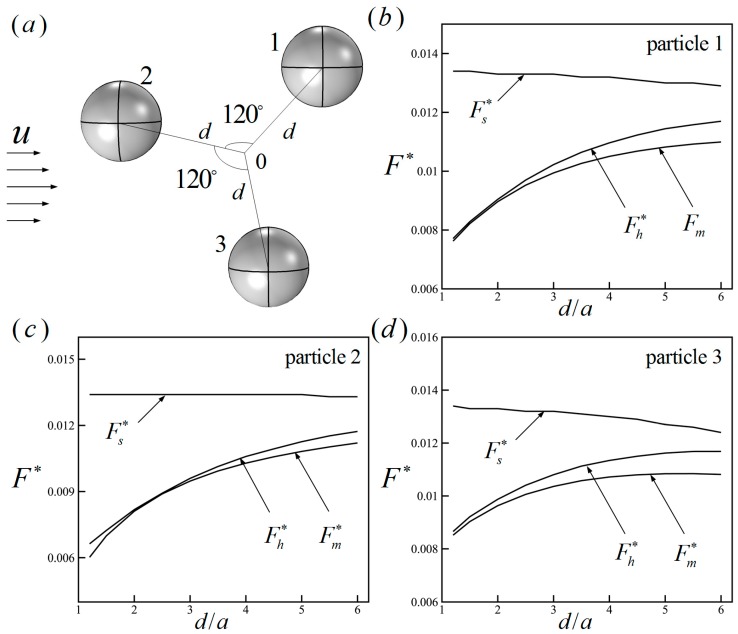
(**a**) The distribution of three particles; (**b**–**d**) the dimensionless hydrodynamic forces versus the ratio of the distance and the particle radius. $F_{h}^{*}$, $F_{s}^{*}$ and $F_{m}^{*}$ are the hydrodynamic forces, respectively calculated by integrating the hydrodynamic stress tensor, the traditional Stokes formula and the modified Stokes formula.

To verify the accuracy of the modified Stokes formula in consideration of near-field hydrodynamic hindrance, the dimensionless hydrodynamic forces $F_{h}^{*}$, $F_{s}^{*}$ and $F_{m}^{*}$, respectively calculated by integrating the hydrodynamic stress tensor, the traditional Stokes formula and the modified Stokes formula, are compared. Three spherical particles (a=5 μm) are suspended in a channel (L×W×H=300 μm×200 μm×200 μm) with a pressure-driven flow (Δp=1 Pa) along the length direction. The three particles are distributed on the plane parallel to the flow direction, as shown in Figure 2a, where *d* is the distance between the particle center and the origin. The dependences of the hydrodynamic forces on the ratio of the distance *d* and the particle radius *a* of the three particles are shown in Figure 2b–d. As we can see, when the particles get closer to each other, the traditional Stokes formula is not applicable for calculating the hydrodynamic force, while the modified Stokes formula can describe the hydrodynamic hindrance accurately. In this paper, the modified Stokes formula is able to calculate the hydrodynamic forces of dense particles.

## 3. Numerical Examples and Discussions

**Figure 3:**
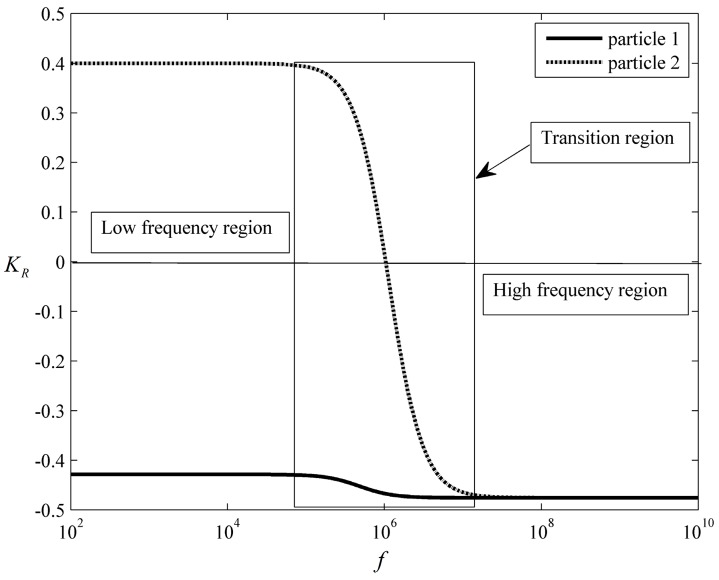
The real parts of the C–M factors of the two types of particles as a function of the frequency. In the low-frequency region, particle 1 is an nDEP particle, while particle 2 is a pDEP particle. In the transition region, particle 2 is transformed from a pDEP particle into an nDEP particle. In the high-frequency region, both particles are nDEP particles.

The fluid and particles in the present example were water and polystyrene beads, respectively. The densities of the particles were the same as the fluid, $\rho =10^{3}\;{\mathrm{kg}/m}^{3}$; thus, the gravity was ignored. The permittivities and conductivities of the fluid were $\varepsilon_{f}=78\varepsilon_{0}$ and $\sigma_{f}=2\times 10^{-3}\;S/m$, respectively, where $\varepsilon_{0}=8.854\times 10^{-12}\;F/m$ was the permittivity in a vacuum. The viscosity of the fluid was $\eta =10^{-3}\;\mathrm{Pa}\cdot s$. The properties of the fluid were specified as the same in all examples of this work. Two types of particles with same permittivities but different conductivities $\varepsilon_{p1}=\varepsilon_{p2}=2.5\varepsilon_{0}$, $\sigma_{p1}=2\times 10^{-4}\;S/m,\;\sigma_{p2}=6\times 10^{-3}\;S/m$ were adopted in this work to study the particle interactions. The real part $K_{R}$ of the C–M factors of the two types of particles versus frequency are shown in Figure 3. As we can see, in the low-frequency region, for particle 1, $K_{R}=-0.4286<0$, suffering a negative DEP force, called an nDEP particle. For particle 2, $K_{R}=0.4>0$, suffering a positive DEP force, called a pDEP particle. In this case, they are called dissimilar particles in this work. In the transition region, particle 2 was rapidly transformed from a pDEP particle into an nDEP particle while particle 1 remained as an nDEP particle. In the high-frequency region, both types of particles were nDEP particles as $K_{R}=-0.4759$. Accordingly, they are called similar particles in the present example.

### 3.1. The Interaction of Five Particles with Different Conductivities

**Figure 4:**
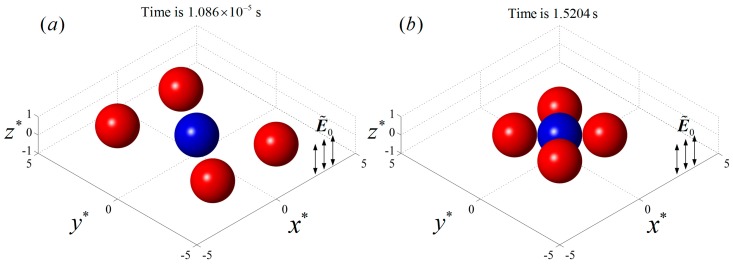
The DEP interaction of five particles initially positioned on a plane perpendicular to an AC electric field with ${\tilde{E}}_{0}=0.02$ and $f=10^{3}\;\mathrm{Hz}$. (**a**) The initial particle locations; (**b**) the final cross-shaped particle chain. The blue and red colors denote pDEP and nDEP particles, respectively.

Five particles with the same size ($a^{*}=1$) were positioned on a plane perpendicular to the uniform AC field ${\tilde{E}}_{0}=0.02$ in the electrolyte. The initial locations of the five particles are listed in Table 1. Four particles were symmetrically distributed about the central particle 1. The permittivities and conductivities of the particles were $\varepsilon_{pi}=2.5\varepsilon_{0},\;i=1,5$ and $\sigma_{p1}=6\times 10^{-3}\;S/m$, $\sigma_{p2}=\sigma_{p3}=\sigma_{p4}=\sigma_{p5}=2\times 10^{-4}\;S/m$, respectively. When a uniform AC field with $f=10^{3}\;\mathrm{Hz}$ was applied, particle 1 behaved as a pDEP particle while the other four particles behaved as nDEP particles, according to Figure 3. The initial particle locations and the final particle chain are shown in Figure 4, where the blue and red colors denote pDEP and nDEP particles, respectively. As we can see, the four nDEP particles were attracted by the central pDEP particle and moved towards the center. Finally the nDEP particles distributed symmetrically around the pDEP particle as the neighboring nDEP particles kept almost the same distance from each other, and a cross-shaped particle chain was formed.

**Figure 5:**
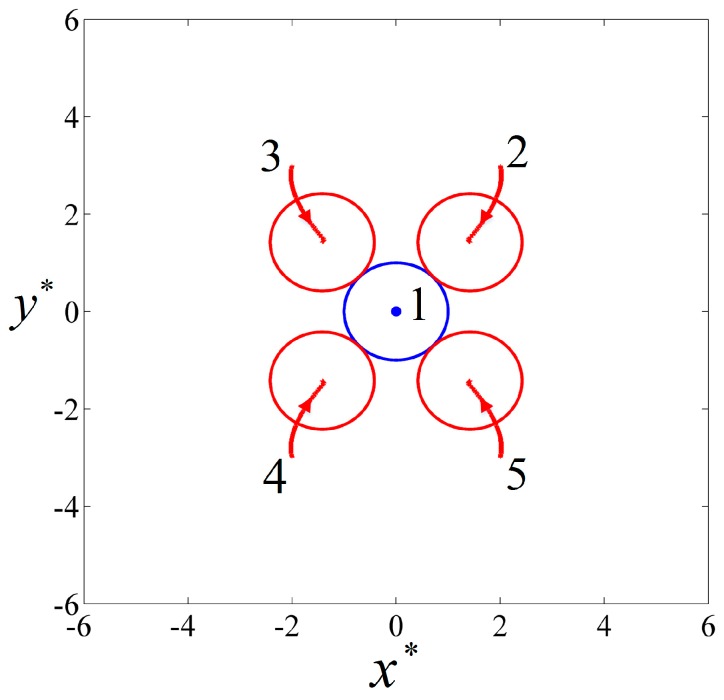
The particle trajectories and the final positions of five particles in a perpendicular AC field with ${\tilde{E}}_{0}=0.02$ and $f=10^{3}\;\mathrm{Hz}$. The blue and red colors denote pDEP and nDEP particles, respectively.

The particle trajectories are shown in Figure 5. It can be seen that all particles moved only on the initial plane in a perpendicular electric field. The central pDEP particle 1 did not move all the time due to symmetrical forces. The nDEP particles 2 and 3 simultaneously suffered attractive forces from particle 1 and repulsive forces from each other. As a result, they moved towards particle 1 while moving away from each other, as did nDEP particles 4 and 5.

These in-plane motions and DEP behaviors can be connected to a special case observed in previous studies. It is well known that similar particles form particle chains parallel to the electric field and dissimilar particles form particle chains perpendicular to the electric field [2]. However, when the particles are initially positioned in the special situation where the connecting line of the particles is perpendicular to the electric field, the similar particles will repel each other while dissimilar particles attract each other. Both the directions of the repulsive force and the attractive force are parallel to their connecting line. In this present 3D model, any two particles are always in the special situation as the electric field is perpendicular to the particle plane. As a result, the interactive forces of the particles are always parallel to their initial plane and they can only move in the plane.

**Figure 6:**
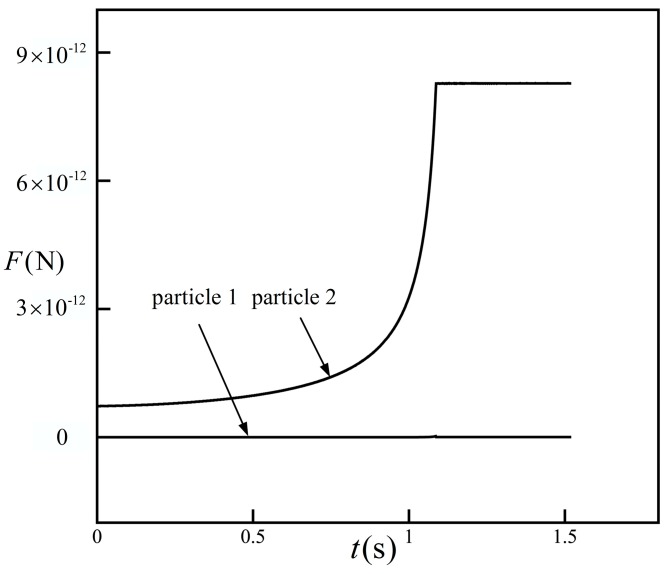
The DEP interactive forces of particle 1 and particle 2 with time. The force of particle 1 is almost zero, the force of particle 2 increases rapidly as particle 2 get closer to particle 1, and finally reaches a constant value when the particle group is formed.

The variations of the DEP interactive forces of particle 1 and particle 2 with time are shown in Figure 6, where the blue color denotes particle 1 and the red color denotes particle 2. It can be seen that the force of particle 1 was zero all the time due to the symmetrical distribution of the other four particles, which is consistent with Figure 5, while the force of particle 2 increased rapidly with time and finally reached a constant value when particle 2 came into contact with particle 1. The DEP interactive forces of particles 3, 4, and 5 are similar to particle 2 and will not be presented.

**Figure 7:**
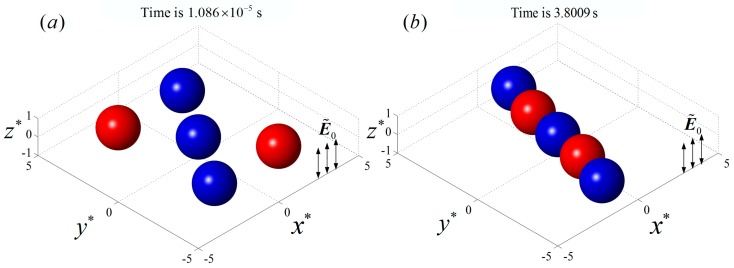
The DEP interaction of five particles with same properties and initial positions as Figure 4, except the conductivities of particle 2 and particle 4 ($\sigma_{p2}=\sigma_{p4}=6\times 10^{-3}\;S/m$) are on a plane perpendicular to a uniform AC electric field with ${\tilde{E}}_{0}=0.02$ and $f=10^{3}\;\mathrm{Hz}$. (**a**) The initial positions of the particles; (**b**) the final line-styled particle chain with alternately arranged pDEP and nDEP particles. The blue and red colors denote pDEP and nDEP particles, respectively.

When the conductivities of particles 2 and 4 were changed to $\sigma_{p2}=\sigma_{p4}=6\times 10^{-3}\;S/m$ and the rest of the parameters remained the same as in the previous example, particles 1, 2, and 4 were pDEP particles and particles 3 and 5 were nDEP particles as shown in Figure 3. The initial locations and the final particle chain are shown in Figure 7, where the blue and red colors denote pDEP and nDEP particles, respectively. It can be seen that the two nDEP particles were respectively attracted by the neighboring two pDEP particles, moving towards the regions between the two pDEP particles. The pDEP particles repelled each other and, finally, an alternately arranged particle chain was formed.

**Figure 8:**
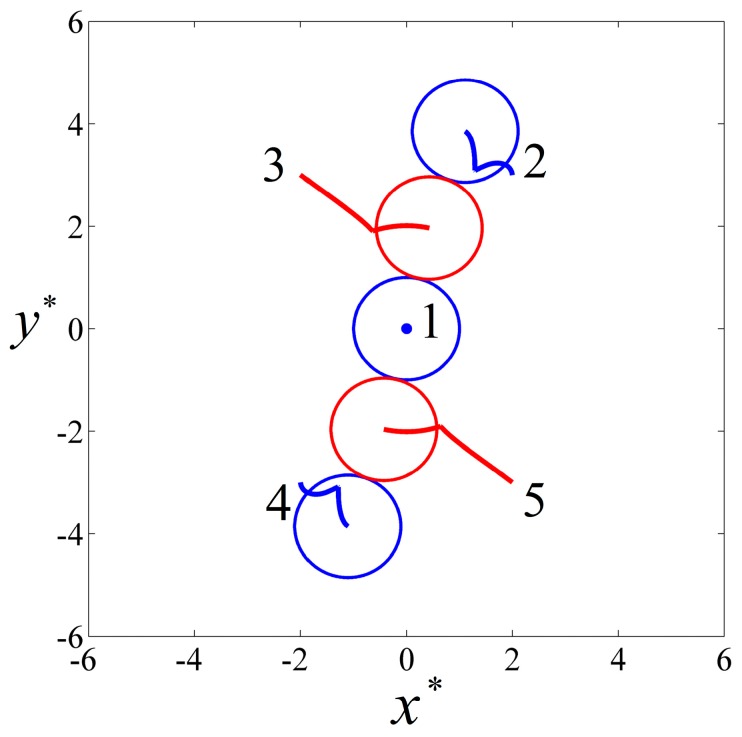
The particle trajectories and the final positions of five particles under a perpendicular AC field with ${\tilde{E}}_{0}=0.02$ and $f=10^{3}\;\mathrm{Hz}$. The blue and red colors denote pDEP and nDEP particles, respectively.

The particles’ trajectories are shown in Figure 8, where the blue and red colors denote pDEP and nDEP particles, respectively. It can be seen that the central particle 1 did not move due to symmetrical forces, while the nDEP particle 3 was attracted by two pDEP particles 1 and 2, moving towards the region between them. Similarly, particle 5 moved towards the region between particles 1 and 4. Finally, a line-styled particle chain consisting of alternating arrangements of nDEP and pDEP particles was formed on the initial plane.

### 3.2. The Interaction of a Large Number of Particles with the Same Size

**Figure 9:**
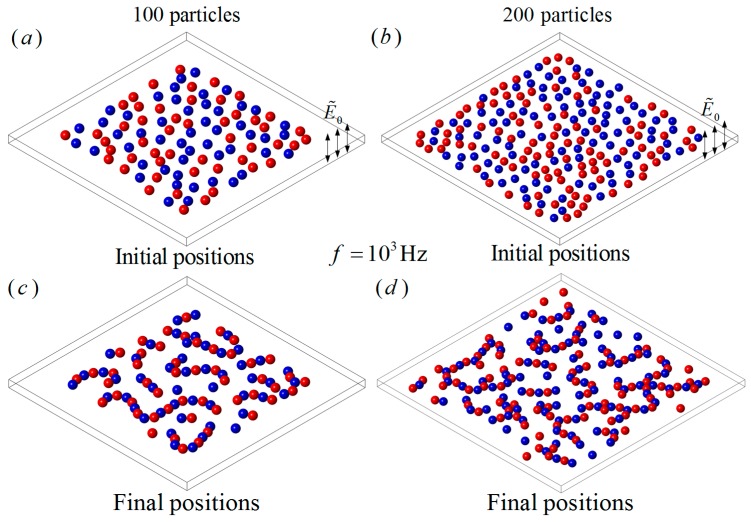
The DEP interaction of a large number of randomly distributed particles under a perpendicular field with ${\tilde{E}}_{0}\;=\;0.02$ and $f=10^{3}\;\mathrm{Hz}$. (**a**) The initial positions of 100 dissimilar particles; (**b**) the initial positions of 200 dissimilar particles; (**c**) particle chains of 100 dissimilar particles; (**d**) particle chains of 200 dissimilar particles. The blue and red colors denote pDEP and nDEP particles, respectively.

One hundred particles with the same size $a^{*}=1$ were randomly distributed on a plane chip filled with electrolyte perpendicular to a uniform AC electric field ${\tilde{E}}_{0}=0.02$, as shown in Figure 9a. The electrical properties of the fluid were the same as that of the previous examples, while 50 particles had the properties of $\varepsilon_{p1}=2.5\varepsilon_{0},\;\sigma_{p1}=6\times 10^{-3}\;S/m$, and the other 50 particles had the properties of $\varepsilon_{p2}=2.5\varepsilon_{0},\sigma_{p2}=2\times 10^{-4}\;S/m$. When $f=10^{3}\;\mathrm{Hz}$, the particles with $\sigma_{p1}=6\times 10^{-3}\;S/m$ behaved as pDEP particles while the others were nDEP particles, according to Figure 3. As we can see in Figure 9c, similar particles repelled each other and dissimilar particles attracted each other and, finally, many particle chains with different styles and lengths were formed, for example line-styled, cross-shaped or a combination of both. Each particle chain consisted of alternately arranged nDEP and pDEP particles. The directions and shapes of the particle chains only depended on their initial distributions and the electric properties.

When the particle number increased to 200, half of the particles had the properties of $\varepsilon_{p1}=2.5\varepsilon_{0},\;\sigma_{p1}=6\times 10^{-3}\;S/m$ and the other half had the properties of $\varepsilon_{p2}=2.5\varepsilon_{0},\sigma_{p2}=2\times 10^{-4}\;S/m$. The initial positions and the final particle chains when $f=10^{3}\;\mathrm{Hz}$ are shown in Figure 9b,d. It can be seen that the particle chain patterns have similar styles but with more complexity, and more branches in the particles chains are observed than in the case of 100 particles.

### 3.3. The DEP Interaction of Large Numbers of Particles on a Bounded Circular Plate Chip

**Figure 10:**
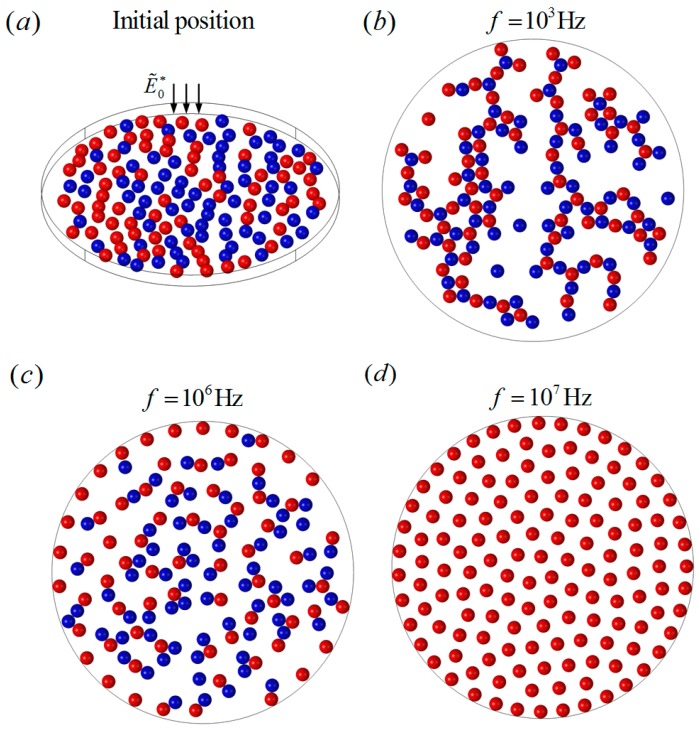
The DEP interactions of randomly distributed particles on a bounded circular plane chip under a perpendicular AC field ${\tilde{E}}_{0}^{*}=0.02$ with different frequencies. (**a**) The initial random distribution of 140 particles; (**b**) the particle chains when $f=10^{3}\;\mathrm{Hz}$; (**c**) the particle chains when $f=10^{6}\;\mathrm{Hz}$; (**d**) the uniform distribution when $f=10^{7}\;\mathrm{Hz}$.

The working chambers of microfluidic chips are all confined spaces, and the chip wall’s effects on DEP particle interactions cannot be neglected in the cases of a larger number of dense particles. DEP particle interactions on a bounded circular plane chip are studied in this section, where 140 particles of uniform size $a^{*}=1$ were randomly distributed on a circular plane chip with a diameter $d^{*}=44$. A uniform AC electric field ${\tilde{E}}_{0}^{*}=0.02$ was applied perpendicularly to the plane chip, as shown in Figure 10a. Half of the particles had the electrical properties of $\varepsilon_{p1}=2.5\varepsilon_{0}$, $\sigma_{p1}=6\times 10^{-3}\;S/m$, and the others had the properties of $\varepsilon_{p2}=2.5\varepsilon_{0}$, $\sigma_{p2}=2\times 10^{-4}\;S/m$. Three cases of frequencies, $f=10^{3}\;\mathrm{Hz}$, $f=10^{6}\;\mathrm{Hz}$, and $f=10^{7}\;\mathrm{Hz}$, were studied in the present work. When $f=10^{3}\;\mathrm{Hz}$, the two groups of particles experienced negative and positive DEP forces, respectively, according to Figure 3. Various particle chains were formed with a more complex structure compared with Figure 9. The scattered short particle chains tended to aggregate with a much larger size and more branches due to the restriction of the boundary, as shown in Figure 10b. When $f=10^{6}\;\mathrm{Hz}$, the particles with $\sigma_{p1}=6\times 10^{-3}\;S/m$ suffered much weaker positive DEP forces, while the others still suffered strong negative DEP forces. The attractive forces between the pDEP and nDEP particles became much weaker, while the nDEP particles still experienced strong repulsive forces between each other. As a result, the particle chains were more scattered compared to those at low frequency, as we can see in Figure 10c. When $f=10^{7}\;\mathrm{Hz}$, all particles behaved as negative DEP particles. It can be expected that all particles experienced strong repulsive forces and moved away from each other, until all the repulsive forces of each particle reached a balance, resulting in an almost uniform particle distribution, finally, as shown in Figure 10d. It was also found that the final particle distributions were quite similar for different random distributions of particles.

**Figure 11:**
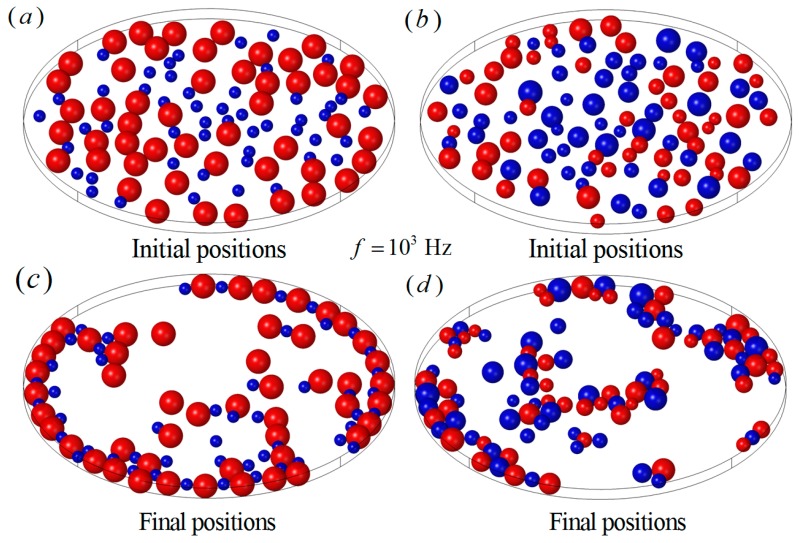
The particle chains with different particle sizes and conductivities when $f=10^{3}\;\mathrm{Hz}$. (**a**) The initial particle positions of two different sizes, half of the particles with radius $a^{*}=1$ and the other half of the particles with radius $a^{*}=2$; (**b**) the initial particle positions of random particle sizes from $a^{*}=1$ to $a^{*}=2$; (**c**) the particle chains of two different sizes; (**d**) the particle chains of random particle sizes.

Particle chain behaviors of different particle sizes and conductivities were also investigated in this work. One hundred particles were split into two groups (50, 50) and the particle properties were specified as $a_{1}^{*}=1$, $\varepsilon_{p1}=2.5\varepsilon_{0}$, $\sigma_{p1}=6\times 10^{-3}\;S/m$ and $a_{2}^{*}=2$, $\varepsilon_{p2}=2.5\varepsilon_{0}$, $\sigma_{p2}=2\times 10^{-4}\;S/m$, respectively, as shown in Figure 11a. The frequency of the electric field was $f=10^{3}\;\mathrm{Hz}$. The rest of the parameters remained the same as in the previous examples. The small particles behaved as positive DEP particles and the large particles behaved as negative DEP particles in the present example. It can be seen that the large particles experienced large repulsive forces between each other and small attractive forces from the small particles. The large particles were repelled from the boundary, while the small particles were attracted by the large particles and, finally, long particle chains along the wall were formed, as shown in Figure 11c. In the case of particles with random radii, from $a^{*}=1$ to $a^{*}=2$, 50 particles with the properties of $\varepsilon_{p1}=2.5\varepsilon_{0}$, $\sigma_{p1}=6\times 10^{-3}\;S/m$ behaved as positive DEP particles, and the other particles with the properties of $\varepsilon_{p2}=2.5\varepsilon_{0}$, $\sigma_{p2}=2\times 10^{-4}\;S/m$ behaved as negative DEP particles, as shown in Figure 11b. The particles were more likely to form clusters than long chains, as shown in Figure 11d.

## 4. Conclusions

The DEP interactions of a large number of dense spherical particles on a plane perpendicular to a uniform AC field were studied by an iterative dipole moment (IDM) model in this work. Numerical examples indicated that the IDM model is an effective method to predict DEP interaction behaviors of a large number of dense spherical particles in an electric field. Some novel phenomena were found in the results and are consistent with the previous studies. When the particles are initially positioned on a plane perpendicular to the electric field, they cannot move out of the plane. Similar DEP spherical particles on a plane perpendicular to an electric field always repel each other, and scatter away. A large number of dense particles in the bounded region eventually form a uniformly distributed particle pattern (equidistant between every two particles) that is almost independent of the initial positions. Dissimilar DEP particles in a plane perpendicular to an electric field always attract each other, forming alternately arranged particle chains, clusters, or groups. The directions and shapes of the final particle chains depend on the particle sizes, number, electrical properties, initial particle distributions, and electric field frequencies. Above all, similar particles and dissimilar DEP particles can easily be transformed into each other via frequency modulation which can be an effective method for particle manipulation in applications of biology, chemistry, and micro-fabrications.

## Figures and Tables

**Table 1:** The initial locations of the five particles.

Coordinate	Particle 1	Particle 2	Particle 3	Particle 4	Particle 5
$x^{*}$	0	2	−2	−2	2
$y^{*}$	0	3	3	−3	−3
$z^{*}$	0	0	0	0	0

## References

[1] Jones T.B. (1995). Electromechanics of Particles.

[2] Morgan H., Green N.G. (2002). AC Electrokinetics: Colloids and Nanoparticles.

[3] Kang Y., Li D. (2009). Electrokinetic motion of particles and cells in microchannels. Microfluid. Nanofluid..

[4] Piacentini N., Mernier G., Tornay R., Renaud P. (2011). Separation of platelets from other blood cells in continuous-flow by dielectrophoresis field-flow-fractionation. Biomicrofluidics.

[5] Moon H.S., Kwon K., Kim S.I., Han H., Sohn J., Lee S., Jung H.I. (2011). Continuous separation of breast cancer cells from blood samples using multi-orifice flow fractionation (MOFF) and dielectrophoresis (DEP). Lab Chip.

[6] Alshareef M., Metrakos N., Perez E.J., Azer F., Yang F., Yang X., Wang G. (2013). Separation of tumor cells with dielectrophoresis-based microfluidic chip. Biomicrofluidics.

[7] Salomon S., Leichlé T., Nicu L. (2011). A dielectrophoretic continuous flow sorter using integrated microelectrodes coupled to a channel constriction. Electrophoresis.

[8] Ben-Bassat D., Boymelgreen A., Yossifon G. (2015). The influence of flow intensity and field frequency on continuous-flow dielectrophoretic trapping. J. Colloid Interface Sci..

[9] Liu W., Shao J., Jia Y., Tao Y., Ding Y., Jiang H., Ren Y. (2015). Trapping and chaining self-assembly of colloidal polystyrene particles over a floating electrode by using combined induced-charge electroosmosis and attractive dipole–dipole interactions. Soft Matter.

[10] Lumsdon S.O., Kaler E.W., Williams J.P., Velev O.D. (2003). Dielectrophoretic assembly of oriented and switchable two-dimensional photonic crystals. Appl. Phys. Lett..

[11] Lumsdon S.O., Kaler E.W., Velev O.D. (2004). Two-dimensional crystallization of microspheres by a coplanar AC electric field. Langmuir.

[12] Gangwal S., Pawar A., Kretzschmar I., Velev O.D. (2010). Programmed assembly of metallodielectric patchy particles in external AC electric fields. Soft Matter.

[13] Giner V., Sancho M., Lee R.S., Martínez G., Pethig R. (1999). Transverse dipolar chaining in binary suspensions induced by RF fields. J. Phys. D Appl. Phys..

[14] Hermanson K.D., Lumsdon S.O., Williams J.P., Kaler E.W., Velev O.D. (2001). Dielectrophoretic assembly of electrically functional microwires from nanoparticle suspensions. Science.

[15] Gupta S., Alargova R.G., Kilpatrick P.K., Velev O.D. (2008). On-chip electric field driven assembly of biocomposites from live cells and functionalized particles. Soft Matter.

[16] Zhou R., Chang H.C., Protasenko V., Kuno M., Singh A.K., Jena D., Xing H. (2007). CdSe nanowires with illumination-enhanced conductivity: Induced dipoles, dielectrophoretic assembly, and field-sensitive emission. J. Appl. Phys..

[17] Schütte J., Hagmeyer B., Holzner F., Kubon M., Werner S., Freudigmann C., Benz K., Böttger J., Gebhardt R., Becker H. (2011). “Artificial micro organs”—A microfluidic device for dielectrophoretic assembly of liver sinusoids. Biomed. Microdevices.

[18] Aubry N., Singh P. (2006). Control of electrostatic particle-particle interactions in dielectrophoresis. Europhys. Lett..

[19] Aubry N., Singh P. (2006). Influence of particle-particle interactions and particles rotational motion in traveling wave dielectrophoresis. Electrophoresis.

[20] Ai Y., Qian S. (2010). DC dielectrophoretic particle–particle interactions and their relative motions. J. Colloid Interface Sci..

[21] Ai Y., Zeng Z., Qian S. (2014). Direct numerical simulation of AC dielectrophoretic particle–particle interactive motions. J. Colloid Interface Sci..

[22] Xie C., Chen B., Ng C.O., Zhou X., Wu J. (2015). Numerical study of interactive motion of dielectrophoretic particles. Eur. J. Mech. B Fluids.

[23] Xie C., Liu L., Chen B., Wu J., Chen H., Zhou X. (2015). Frequency effects on interactive motion of dielectrophoretic particles in an AC electrical field. Eur. J. Mech. B Fluids.

[24] Kang S., Maniyeri R. (2012). Dielectrophoretic motions of multiple particles and their analogy with the magnetophoretic counterparts. J. Mech. Sci. Technol..

[25] Kang S. (2013). Two-dimensional dipolophoretic motion of a pair of ideally polarizable particles under a uniform electric field. Eur. J. Mech. B Fluids.

[26] Kang S. (2014). Dielectrophoretic motion of two particles with diverse sets of the electric conductivity under a uniform electric field. Comput. Fluids.

[27] Kang S. (2015). Dielectrophoretic motions of multiple particles under an alternating-current electric field. Eur. J. Mech. B Fluids.

[28] Hossan M.R., Dillon R., Roy A.K., Dutta P. (2013). Modeling and simulation of dielectrophoretic particle–particle interactions and assembly. J. Colloid Interface Sci..

[29] Kretschmer R., Fritzsche W. (2004). Pearl chain formation of nanoparticles in microelectrode gaps by dielectrophoresis. Langmuir.

[30] Sancho M., Martínez G., Muñoz S., Sebastián J.L., Pethig R. (2010). Interaction between cells in dielectrophoresis and electrorotation experiments. Biomicrofluidics.

[31] Lee D.H., Yu C., Papazoglou E., Farouk B., Noh H.M. (2011). Dielectrophoretic particle–particle interaction under AC electrohydrodynamic flow conditions. Electrophoresis.

[32] Zhao Y., Hodge J., Brcka J., Faguet J., Lee E., Zhang G. Effect of electric field distortion on particle-particle interaction under DEP. Proceedings of the COMSOL Conference 2013.

[33] Liu L., Xie C., Chen B., Wu J. (2015). Iterative dipole moment method for calculating dielectrophoretic forces of particle-particle electric field interactions. Appl. Math. Mech..

[34] Liu L., Xie C., Chen B., Chiu-On N., Wu J. (2015). A new method for the interaction between multiple DEP particles: Iterative dipole moment method. Microsyst. Technol..

[35] Liu L., Xie C., Chen B., Wu J. (2015). Numerical study of particle chains of a large number of randomly distributed DEP particles using iterative dipole moment method. J. Chem. Technol. Biotechnol..

[36] Xie C., Chen B., Liu L., Chen H., Wu J. (2016). Iterative dipole moment method for the interaction of multiple dielectrophoretic particles in an AC electrical field. Eur. J. Mech. B Fluids.

[37] White F.M., Corfield I. (2006). Viscous Fluid Flow.

